# Improving the Quantification of Colorimetric Signals in Paper-Based Immunosensors with an Open-Source Reader

**DOI:** 10.3390/s22051880

**Published:** 2022-02-27

**Authors:** Steven M. Russell, Alejandra Alba-Patiño, Andreu Vaquer, Antonio Clemente, Roberto de la Rica

**Affiliations:** 1 Multidisciplinary Sepsis Group, Health Research Institute of the Balearic Islands (IdISBa), 07120 Palma de Mallorca, Spain; francyalejandra.alba@ssib.es (A.A.-P.); andreu.vaquer@ssib.es (A.V.); roberto.delarica@ssib.es (R.d.l.R.); 2 Chemistry Department, University of the Balearic Islands, 07122 Palma de Mallorca, Spain; 3 CIBER de Enfermedades Infecciosas (CIBERINFEC), 28029 Madrid, Spain

**Keywords:** lateral flow test, COVID-19, immunosensor, biosensor, open-source, image processing, rapid test reader

## Abstract

Measuring the colorimetric signals produced by the biospecific accumulation of colorimetric probes and recording the results is a key feature for next-generation paper-based rapid tests. Manual processing of these tests is time-consuming and prone to a loss of accuracy when interpreting faint and patchy signals. Proprietary, closed-source readers and software companies offering automated smartphone-based assay readings have both been criticized for interoperability issues. Here, we introduce a minimal reader prototype composed of open-source hardware and open-source software that has the benefits of automatic assay quantification while avoiding the interoperability issues associated with closed-source readers. An image-processing algorithm was developed to automate the selection of an optimal region of interest and measure the average pixel intensity. When used to quantify signals produced by lateral flow immunoassays for detecting antibodies against SARS-CoV-2, results obtained with the proposed algorithm were comparable to those obtained with a manual method but with the advantage of improving the precision and accuracy when quantifying small spots or faint and patchy signals.

## 1. Introduction

Colorimetric paper-based devices have become crucial tools for managing infectious diseases by allowing the rapid immunodetection of antigens, host antibodies, and inflammation biomarkers at the point of care (POC) [1,2,3,4,5,6]. The key to this success lies in combining a paper-based microfluidic system with the outstanding specificity of antibody recognition elements and a signal transduction mechanism that can be interpreted by eye [7,8]. The latter is commonly achieved by using gold nanoparticles as colorimetric probes [9]. The naked-eye detection scheme maximizes the portability and cost-effectiveness of the analytical platform. However, naked-eye detection also comes with limitations. Faint color changes on the test strip can be nearly indistinguishable from the background substrate. This is especially problematic for POC tests since different settings may have incomparable lighting environments that affect the interpretation of results. This means that samples that are close to the limit of detection may be miscategorized [10,11]. Additionally, evidence suggests that results from point-of-care tests often go unrecorded, and even when results are recorded, they tend to be qualitative and unstandardized [12]. Measuring the colorimetric signal produced by the biospecific accumulation of colorimetric probes and recording the results can overcome these limitations [13]. In many laboratories, this is achieved with reflective densitometry. This is typically performed by imaging the test, manually selecting the region of interest (ROI) within the colored spot, and measuring the average pixel intensity of a responsive color channel [11,14,15,16]. This approach, however, is too laborious and time-consuming for rapid mass testing schemes, and it is prone to human error when selecting the ROI, which reduces the accuracy of the measurements.

**Figure 1:**
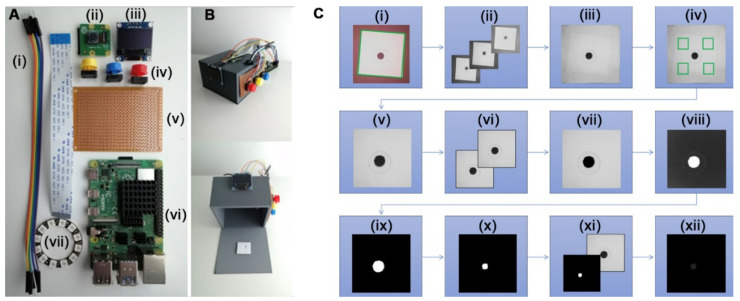
Reader hardware and software for automated quantification of colorimetric signals in paper-based immunosensors: (**A**) Reader components, (**i**) connecting cables, (**ii**) image sensor, (**iii**) display screen, (**iv**) push buttons, (**v**) solder board, (**vi**) single board computer, (**vii**) LED ring; (**B**) Photographs of the reader mounted on an 8 × 8 × 5 cm 3D-printed box with a detached bottom panel; (**C**) Image capture and post-processing algorithmic workflow: (**i**) image capture upon edge detection, (**ii**) split RGB channels, (**iii**) crop outer edge of G channel image, (**iv**) measure background, (**v**) center crop and resize, (**vi**) duplicate, (**vii**) threshold, (**viii**) invert, (**ix**) binarize for mask, (**x**) dilate and erode mask, (**xi**) apply mask to duplicate, (**xii**) measure ROI.

In this article, we introduce an open-source solution that automatically performs all the image processing steps required for accurate signal quantification in paper-based biosensors. The software was developed with a minimal reader prototype composed of open-source hardware electrical components (Figure 1A). As a result of the open design, anyone can make a copy of the device and modify it for their own purposes, enabling community-driven development based on FAIR principles. The open reader design also allows for new possibilities for rapid fabrication in mass-testing schemes, with streamlined interoperability [17,18]. When tested on printed calibrators, measurements obtained with the proposed reader showed a good correlation with those obtained with a traditional densitometry scheme based on manually selecting the ROI and were more accurate than the manual approach for small signals. When tested on commercial lateral flow tests for measuring antibodies against SARS-CoV-2, the reader gave highly reproducible results, even when the sensor produced patchy signals that yielded disparate results with the manual method.

## 2. Materials and Methods

### 2.1. Hardware

**Figure 2:**
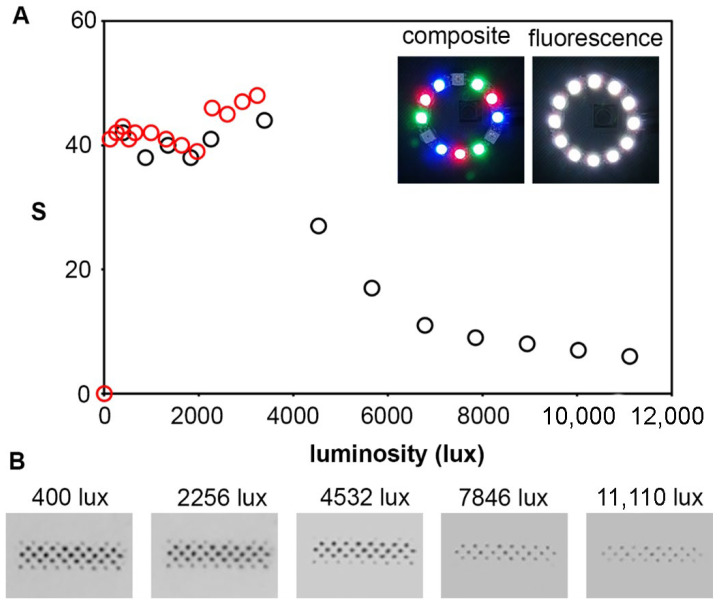
Impact of luminosity in imaging conditions using composite white light with and without fluorescence (shown in inset): (**A**) Impact of light intensity (lux) on densitometric signal S with fluorescence (black) and without fluorescence (red); (**B**) Photographs of the same calibrator taken with fluorescence at different luminosity values.

Wire jumper cables, a 38 mm ring of WS2812B 12-bit LEDs, and a 128 × 64 Pixel I2C OLED display module with a SSD1306 chip were purchased from AZ-Delivery Microelectronics. Pushbuttons, button caps, and resistors were purchased from ELEGOO Electronics. A Pi Camera V2.1 based on the 8MP Sony IMX219 image sensor and a Raspberry Pi 4 model B from the Raspberry Pi foundation were also purchased. The pushbuttons were soldered to a Onogal Model 4112 PCB prototype board and connected to the Raspberry Pi general input/output (GPIO) pins. A 3D box with a white square detection area and small slots for cables and the camera lens was designed in-house. The box has one detached panel on the bottom for loading a paper-based test for measurement. The 3D printing resin for the box is neutral gray, similar to an 18% gray card used in photography, and it is 3 mm thick to prevent light artifacts in the image capture step. The box also serves to align the test with respect to the lights and camera (Figure 1B). The hardware components were arranged on the 3D-printed box that also serves to stabilize the photographic conditions (Figure 1A,B). A printable calibrator set was also developed in-house. Circular-shaped and rectangular-shaped calibrators were designed to approximate the kinds of colorimetric signals found in paper-based colorimetric devices and were printed with an HP 4500 series printer on Torrascontrast high-quality printer paper. The size and shape of each calibrator were printed in a series of step-wise grayscale intensities from 0 (black) to 225 (almost white). Two sizes of circular shapes (3 mm and 1 mm diameters) and two sizes of rectangular shapes (5 × 1.5 mm, and 3 × 0.5 mm) were assayed for each grayscale intensity. The LED ring was configured to an optimal luminosity and composite white light for signal measurement. Calibration details are shown in Figure 2.

### 2.2. Software

Open-source drivers and scripts that accompany the hardware components were downloaded and installed on the Raspberry Pi 4 via Github. The OLED is run with the python package “adafruit_CircuitPython_ssd1306”. The LED ring runs with “rpi-ws281x”. The camera interface is facilitated by the PiCamera API. Communication from the buttons to Raspberry Pi are handled through the python package “RPi.GPIO 0.7.0”. Other open-source licensed code was also used for functions related to image pre-processing [19,20,21]. All software packages hold open-source licenses. The post-processing algorithm was developed in-house, with the OpenCV computer vision library and the PiCamera API. Custom button call-backs and a shell program that initiates the user interface upon device start-up were also developed in-house. A Jupyter notebook detailing the algorithmic steps for image processing and signal quantification, as well as all source code, is available in the repository of supplemental information available at https://github.com/SMR-83/Open_Reader (accessed on 20 February 2022).

The example scripts associated with the hardware components were modified to calibrate the illuminant with respect to the image sensor. The preprocessing script pairs with the hardware via a white paper square on the detached panel of the box that acts as a detection area. The position of the sample with respect to the camera is regularized and quality-checked using the edges of the white square as a reference to align the digital images. Sets of calibration images and lateral flow test images were acquired in this way.

### 2.3. System Integration and User Interface

Upon hitting the blue button, scripts that control the hardware for the image capture step are called. The lights and camera turn on, and image capture starts by searching for the edges of a white paper square “detection area” on the bottom panel. When the camera finds the square, the color channels are split, and the green channel of the image is aligned using the edges of the detection area as a guide, as shown in Figure 1C(i–iii). A message is displayed on the screen to inform the user if the image capture is successful or not. The yellow button calls a post-processing algorithm to measure captured images. The post-processing algorithm measures the background substrate and then crops the edges and scales the image so that signals are more uniformly centered, as shown in Figure 1C(iv,v). The image is then duplicated. One duplicate remains unchanged, and the other is processed into a mask. By algorithmically deriving a mask based on the information contained in the image, the background measurement from before is used as a thresholding limit. This causes all pixels below that limit to be changed to black, as shown in Figure 1C(vi,vii). The thresholding yields an initial segmentation of the signal area from the background. However, the masking process requires a binary black and white image in order to make an ROI. White areas of the mask become transparent when applied to another image, while black areas block out pixel information. As a result, the mask is inverted and binarized, as shown in Figure 1C(viii,ix). The mask is then refined using erosion and dilation operations so that noise is reduced, and more homogeneous regions are selected for the ROI. When the mask is applied to the unchanged duplicate image, the ROI is visible while everything else has a pixel intensity of zero, as shown in Figure 1C(x–xii). The number of pixels that compose the signal is calculated using an operation that counts all non-zero pixels, that is, all non-black pixels after the mask is applied. The pixel intensity of the ROI is calculated by taking the sum of the pixels in the image and dividing by the number of non-zero pixels. The result is displayed on the screen, or an error message is displayed in the event of an unreadable signal. The red button turns the device off.

### 2.4. LFIA Analysis of Plasma Sample

A plasma sample from a volunteer who had been recently vaccinated against SARS-CoV-2 was serially diluted from 1:2 to 1:128 in phosphate buffer saline (PBS) in order to generate samples with different concentrations of antibodies against the virus. The samples were tested with lateral flow immunoassays (LFIA) for COVID-19 IgG/IgM using rapid test cassettes from SureScreen Diagnostics. Each LFIA was imaged and measured three times in order to determine the error associated to the measurement. A plasma sample diluted 1:2 in PBS from an unvaccinated volunteer obtained before the COVID-19 pandemic was used as negative control in order to define the instrumental limit of detection (above 3 times the standard deviation of the control). Images of the tests were acquired with the reader and measured manually and automatically. Manual measurement for solid test lines was performed by selecting an ROI inside the signal avoiding the edges, resulting in rectangular or circular areas slightly smaller than the test line. The mean pixel intensity within the ROI was calculated with the histogram function of ImageJ. Manual measurement of faint and patchy test lines posed a question of what constitutes an ROI that is most representative of the signal. The colorimetric signal S is the average pixel intensity of the ROI.

## 3. Results and Discussion

The brightness of the LED ring (Figure 1A(vii)) was optimized to avoid signal bleaching. White light from the LEDs is a composite of red, green, and blue light. Phosphor-covered LEDs can also be added to the light composition to achieve brighter white light with fluorescence (shown in Figure 2A, inset). The luminosity of each type of white light at step-wise intensities was measured with a lux meter. As shown in Figure 2A, both types of lighting yielded similar results when tested on a lightly colored spot with the luminosity ranging between 100 and 3500 lux. The range between 2000 and 3500 lux yielded slightly higher values without fluorescence. As a result, composite white light near the upper end of the range was chosen as the optimal light setting. Luminosity values higher than 3500 are only achievable with fluorescence and result in an exponential decrease in the colorimetric signal. Photographs in Figure 2B show the decrease in signal caused by sensor saturation. Subsequent experiments were performed with light at approximately 3236 lux.

**Figure 3:**
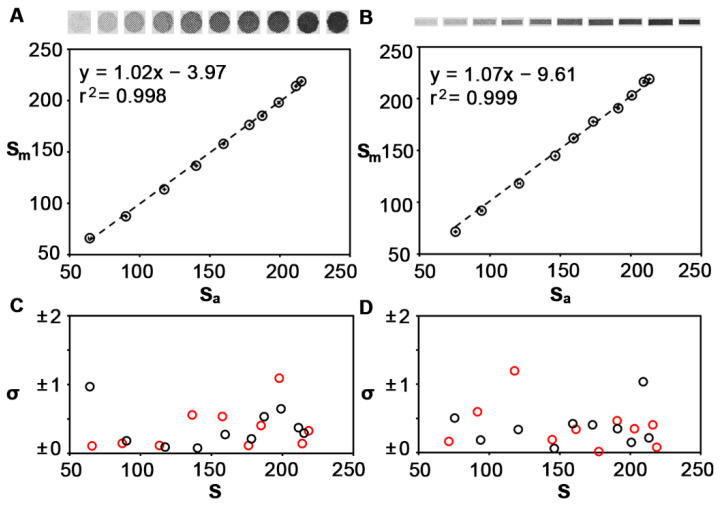
Comparison of larger signals obtained using the manual method (Sm) and the automated method (Sa) for quantifying the pixel intensity. Correlation plots when the spots are shaped like a large circle (**A**), or a large rectangle (**B**), circles are the mean and error bars are the standard deviation (*n* = 3). Standard deviation (σ) associated with colorimetric signals S obtained with the proposed software (black) or with the manual method (red) (**C**,**D**).

Using the reader shown in Figure 1B to capture images, we compared automated signal quantification against the manual method. Colorimetric signals in paper-based biosensors are usually circular (e.g., dot blots) or rectangular (e.g., lateral flow immunoassays). With this in mind, we printed circular and rectangular patterns of increasing color intensity and measured them with both methods (Figure 3A,B). Measurements were performed in triplicate with the same printed calibrators in order to determine the instrumental error. Figure 3A,B show that there is a linear correlation between both methods when the circles have a diameter of 3 mm and the rectangles have dimensions of 5 × 1.5 mm. The slope of the linear regression and the Pearson correlation coefficient are very close to one in both cases, which indicates an almost perfect correlation between both methods. The relative error (standard deviation) was always smaller than one regardless of the method used to perform the measurements, and no trend was observed with regards to the color intensity (Figure 3C,D). This results in minuscule error bars, located within the circular data points in Figure 3A,B. These experiments demonstrate that the proposed software compares well with the manual signal quantification method when measuring large colored spots. Yet, the former only requires pushing a button and waiting a few seconds, whereas the latter requires trained personnel and takes several minutes to yield an answer.

**Figure 4:**
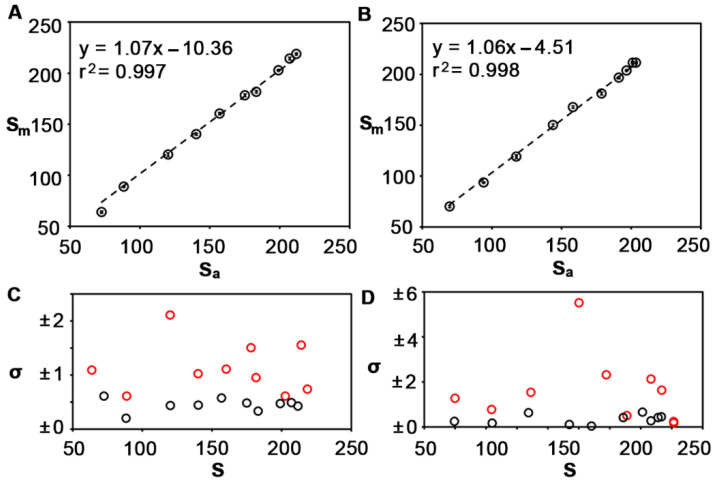
Comparison of smaller signals obtained using the manual method (S_m_) or the automated method (S_a_) for quantifying the pixel intensity. Correlation plots when the spots are shaped like a small circle (**A**), or a small rectangle (**B**), circles are the mean and error bars are the standard deviation (*n* = 3). Standard deviation (σ) associated with colorimetric signals S obtained with the proposed software (black) or with the manual method (red) (**C**,**D**).

Figure 4A,B show that there is still a strong linear correlation between both methods when the dimensions of the circle and the rectangle are reduced to 1 mm diameter and 3 mm × 0.5 mm, respectively. However, the standard deviation of measurements performed with the software was never higher than one, whereas it often exceeded this value when the measurements were performed with the manual method (Figure 4C,D). That is, the manual method is less accurate when measuring small signals. This is ascribed to the lower number of pixels in smaller objects. In large spots, selecting different regions of interest manually does not change the measured colorimetric signal significantly because discrepancies are reduced when calculating the average pixel intensity. Smaller spots have fewer pixels and do not average out differences as well as their larger counterparts. These experiments demonstrate that results obtained with the algorithm are comparable to those obtained with a manual method but with the advantage of improving the precision when quantifying smaller colored spots.

**Figure 5:**
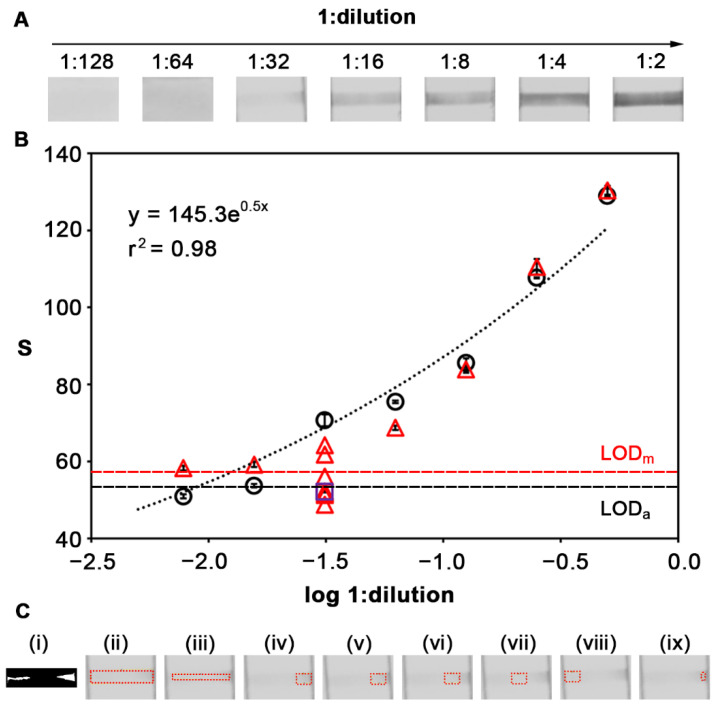
Colorimetric detection of LFIA for host antibodies against SARS-CoV-2: (**A**) Photographs of the assays; (**B**) signal quantification with the automated software (black dots) or the manual method (red triangles and purple square), where error bars are the standard deviation of 3 measurements on the same LFIA, LOD_m_ is the limit of detection obtained with manual method, and LOD_a_ is the one obtained with the automated software; (**C**) ROI for the 1:32 samples obtained with the automated software (**i**) or through manual selection that gives rise to the different results reported for sample 1:32 (**ii**–**ix**).

After testing the performance of the reader for detecting the pixel intensity in printed patterns, we sought to test its adequacy for quantifying a real colorimetric biosensor. To this end, LFIAs were used for detecting antibodies (IgG and IgM) against SARS-CoV-2 in serially diluted plasma samples. LFIA test controls were also performed with an undiluted (or diluted 1:2) plasma sample from healthy, vaccinated volunteers to demonstrate the presence of IgG antibodies against SARS-CoV-2 (IgM antibodies were not detected). Control image datasets are available in the Github repository. Figure 5A shows photographs of the colorimetric signals yielded by IgG anti-SARS-CoV-2 antibodies after serial plasma dilutions. The test line of each LFIA was imaged three times with the reader and measured with the manual method and the automated algorithm. In Figure 5B, there is an exponential relation between the inverse of the dilution factor and the colorimetric signal measured with the algorithm. The limit of detection based on the 3σ criterion using a control plasma sample without antibodies was 1:64. The analysis of the same samples with the manual method yielded very similar results when the concentration of antibodies was high (dilutions 1:8 to 1:2). However, the highly diluted 1:32 sample yielded a patchy signal whose colorimetric signal greatly varied depending on how the ROI was selected, which is shown in Figure 5C. Figure 5C(i) shows the segmentation step performed by the automated software in order to select the ROI. Figure 5C(ii) shows the ROI selected in the same way as it was selected in less diluted samples. Calculating the pixel intensity in this region yielded the signal highlighted as a purple square in Figure 5B, which is below the limit of detection. When choosing an ROI manually, there are more factors to consider. Whether only homogenously colored regions (Figure 5C(ix)), regions where the signal is a gradient (Figure 5C(iv–viii)), or a central region with both colored and uncolored areas (Figure 5C(iii)) are chosen, the ROI yielded different results that were sometimes above or below the limit of detection (red triangles in Figure 5B). In other words, the manual method risks yielding a false positive or false negative result, depending on how the signal is measured. These experiments demonstrate that algorithmically deriving an optimal ROI increases the accuracy of densitometric analysis by removing the human error associated with manually selecting an ROI in faint and patchy signals.

## 4. Conclusions

In conclusion, an algorithm that automatically performs all the steps required for quantifying the pixel intensity in colorimetric paper-based immunosensor was introduced with a minimal reader. Results obtained with the automated method correlated well with the traditional approach based on manually selecting the region of interest and calculating the mean pixel intensity within it. Yet, the automated method was more advantageous in that it increased the precision when measuring small colored spots and the accuracy when quantifying incomplete and faint signals. This granted the reliable measurement of colorimetric signals that were close to the limit of detection when analyzing an LFIA for human anti-SARS-CoV-2 antibodies. Open-source image processing software such as this, combined with a minimal reader prototype comprised of open-source hardware, makes community-driven development possible. A key advantage of this approach is that it can avoid interoperability issues intrinsic to closed-source smartphone apps and commercial readers. A repository of image datasets, hardware specifications, and source-code is openly available at https://github.com/SMR-83/Open_Reader (accessed on 20 February 2022).

## Data Availability

The data presented in this study are openly on Github at doi:10.5281/zenodo.5837440.
